# Changes in mitochondrial thymidine metabolism and mtDNA copy number during induced pluripotency

**DOI:** 10.1038/s12276-025-01476-3

**Published:** 2025-06-26

**Authors:** Hyun Kyu Kim, Yena Song, Minji Kye, Byeongho Yu, Hyung Kyu Choi, Sung-Hwan Moon, Man Ryul Lee

**Affiliations:** 1 Soonchunhyang Institute of Medi-bio Science, Soon Chun Hyang University, Cheonan, Republic of Korea; 2 Dementia Research Group, Korea Brain Research Institute, Daegu, South Korea; 3 Department of Animal Science and Technology, Chung-Ang University, Anseong, Republic of Korea; 4 Department of Stem Cell and Regenerative Biotechnology, KU Institute of Science and Technology, The Institute of Advanced Regenerative Science, Konkuk University, Gwangjin-gu, Republic of Korea

**Keywords:** Induced pluripotent stem cells, Reprogramming

## Abstract

Somatic cell reprogramming into human induced pluripotent stem cells entails significant intracellular changes, including modifications in mitochondrial metabolism and a decrease in mitochondrial DNA copy number. However, the mechanisms underlying this decrease in mitochondrial DNA copy number during reprogramming remain unclear. Here we aimed to elucidate these underlying mechanisms. Through a meta-analysis of several RNA sequencing datasets, we identified genes responsible for the decrease in mitochondrial DNA. We investigated the functions of these identified genes and assessed their regulatory mechanisms. In particular, the expression of the thymidine kinase 2 gene (*TK2*), located in the mitochondria and required for mitochondrial DNA synthesis, is decreased in human pluripotent stem cells as compared with its expression in somatic cells. TK2 was significantly downregulated during reprogramming and markedly upregulated during differentiation. Collectively, this decrease in TK2 levels induces a decrease in mitochondrial DNA copy number and contributes to shaping the metabolic characteristics of human pluripotent stem cells. However, contrary to our expectations, treatment with a TK2 inhibitor impaired somatic cell reprogramming. These results suggest that decreased TK2 expression may result from metabolic conversion during somatic cell reprogramming.

## Mitochondrial DNA loss linked to stem cell reprogramming

Induced pluripotent stem (iPS) cells are special cells created by reprogramming regular body cells. Researchers explored how these cells change their energy production methods during reprogramming. The study focused on a protein called thymidine kinase 2 (TK2), which is important for maintaining mitochondrial DNA (mtDNA). Mitochondria are the cell’s powerhouses, and their DNA is crucial for energy production. Researchers used human cell lines to study how TK2 affects mtDNA during reprogramming. They found that, as cells become iPS cells, TK2 levels drop, leading to reduced mtDNA and a shift in energy production from oxidative phosphorylation to glycolysis. Results suggest that reducing TK2 and mtDNA is key for cells to gain pluripotency. This shift helps support the rapid growth and development of iPS cells. Understanding this process could improve stem cell therapies and regenerative medicine in the future.

This summary was initially drafted using artificial intelligence, then revised and fact-checked by the author.

## Introduction

Induced pluripotent stem (iPS) cells are reprogrammed cells obtained by introducing reprogramming factors (OCT4, SOX2, KLF4 and c-MYC) into somatic cells; this causes the cells to go through the stochastic and determinate phases to acquire pluripotency^1–4^. Reprogramming induces dynamic shifts in gene expression profiles, epigenetic landscapes, metabolic traits and cellular morphology within somatic cells^5,6^. Although comprehensive investigations have been carried out, the acquisition of pluripotency by somatic cells during the stochastic phase is reported to be a slow and inefficient process owing to its intricate nature^2^. Efforts have been made to understand the intricacies of reprogramming; however, the underlying metabolic mechanisms pivotal for acquiring pluripotency remain largely unknown.

To ensure essential energy supply for cell survival, cells typically break down glucose into pyruvate in the presence of oxygen, subsequently subjecting it to oxidative phosphorylation (OXPHOS) within the mitochondria for ATP synthesis. However, even in the presence of sufficient oxygen, pluripotent stem (PS) cells engage in aerobic glycolysis, converting pyruvate into lactate instead of transporting it to the mitochondria for ATP production. This process is relatively inefficient from an energy production perspective. However, in cases where the metabolic flux is sufficiently high, and during the rapid cellular proliferation that characterizes undifferentiated embryonic stem (ES) cells and iPS cells, aerobic glycolysis can be considered a favorable metabolic mechanism^7^. Aerobic glycolysis can result in a rapid supply of ATP and is recognized as a pivotal process that provides essential building blocks, including nucleic acids generated through the pentose phosphate pathway, amino acids and lipids required for biosynthesis, particularly during the robust proliferation observed at the initial stages of proliferation. Therefore, the process by which somatic cells acquire pluripotency through reprogramming involves an essential concomitant transition from the predominant somatic cell metabolism, characterized by OXPHOS, to the metabolic profile characteristic of PS cells, that is, aerobic glycolysis^8–11^. During reprogramming, the fundamental mechanisms underlying metabolic changes have been reported to involve the upregulation of glycolytic genes, which contribute to the activation of this process and concurrently downregulate mitochondrial respiratory chain complex genes to enhance reprogramming efficiency^8–14^. In addition, morphological alterations in the mitochondria influence reprogramming efficiency^10,11,15–17^. During reprogramming, mature tubular and crista-rich mitochondria found in somatic cells transition into immature spherical mitochondria with reduced cristae upon acquisition of pluripotency^16,18,19^. These morphological and functional changes in mitochondria are closely associated with the acquisition of metabolic traits in PS cells and directly affect reprogramming efficiency^15–18,20^.

Of note, PS cells, characterized by aerobic glycolysis, exhibit a marked decrease in mitochondrial DNA (mtDNA) copy number as compared to somatic cells^10,21,22^. mtDNA, a circular DNA molecule spanning 16.6 kb, encodes 13 essential protein subunits of mitochondrial respiratory chain complexes I, III, IV and V, as well as 22 tRNAs and 2 rRNAs required for the translation of mitochondrial subunits^23,24^. Therefore, changes in mtDNA copy number can directly affect mitochondrial function and the acquisition of pluripotency during reprogramming. The regulation of mtDNA copy number is tightly controlled by nuclear-encoded mtDNA replication factors^25^. Loss of function of genes involved in mtDNA replication and synthesis leads to decreased mtDNA copy number, mitochondrial defects and increased reliance on aerobic glycolysis, in a similar way as with the Warburg effect^26^. Similarly, metabolic conversion from OXPHOS to glycolysis occurs during somatic cell reprogramming and is accompanied by a decrease in mtDNA copy number^10,22^. Furthermore, human ES cells have a relatively low number of mtDNA copies as compared with somatic cells^16^. Contrastingly, mitochondria undergo significant changes during cellular differentiation, leading to an increase in mtDNA copy number and the occurrence of a mature structural state characterized by a dense matrix, complex cristae and dispersed localization in the cytoplasm^16–19^. This suggests that mtDNA copy number is closely associated with the identity of stem cells during induced pluripotency. However, the molecular mechanisms underlying alterations in mitochondrial function and mtDNA copy number are not well understood. In this study, we focus on the differential expression of thymidine kinase 2 (TK2) in somatic cells and PS cells, evaluating its potential role in regulating mtDNA copy number and the acquisition of pluripotency characteristics. Human cells possess two enzymes (TK1 and TK2) involved in the phosphorylation of thymidine, a DNA building block necessary for DNA replication and synthesis. TK1 exhibits cell cycle-dependent activity in the cytoplasm, with high activity in rapidly dividing cells, including stem cells and tumor cells; however, it is ubiquitinated and degraded in slow-dividing cells or cells with less active DNA replication^23^. By contrast, TK2, encoded by a gene located on chromosome 16, is translated and targeted to the mitochondria, where it functions independently of the cell cycle and is involved in mitochondrial thymidine synthesis^10,20,21^. This distinction in the roles of TK1 and TK2 suggests a unique metabolic regulation in PS cells and in their mitochondrial function.

This study aimed to explore the role of TK2 in mtDNA synthesis and pluripotency. We investigated TK2 expression levels during the reprogramming process and identified potential TK2-related genetic regulators that influence mtDNA copy number. The insights provided in this study will help to further our understanding of the metabolic characteristics essential for maintaining stemness and enhancing reprogramming efficiency in PS cells.

## Material and methods

### Culture and maintenance of hES cells

The human (h)ES cell lines, H1 and H9 (WiCell Research Institute, Madison, WI, USA), and the hiPS cell lines, CMC-hiPSC-003, 009 and 011 (Korea Centers for Disease Control and Prevention, Osong, Korea), were maintained in complete TeSR-E8 medium (STEMCELL Technologies) for feeder-free culture. PS cells were plated on dishes coated with vitronectin (Vitronectin XF, STEMCELL Technologies, cat. no. 07180). The medium was replaced every 24 h. For passaging, the cells were enzymatically detached using TrypLE Express (Gibco) and transferred to a new coated dish every 5 days. Passages were prepared at split ratios of 1:5 or 1:10. The fibroblast cell lines, BJ1, MRC5 and NT2 (human embryonal carcinoma stem cells), were maintained in Dulbecco’s modified Eagle medium (DMEM, high glucose; Corning) supplemented with 10% fetal bovine serum (Corning) and 100× penicillin–streptomycin (Corning). Fibroblasts and NT2 cells were routinely passaged every 4–5 days using TrypLE Express. All cells were maintained in an incubator at 37 °C under 5% CO_2_.

### Induction of spontaneous in vitro differentiation through EB generation

For embryoid body (EB) generation, clumps of undifferentiated hES cells were mechanically detached using glass pipettes. Subsequently, these clumps were seeded onto nonadhesive bacterial dishes in differentiation medium devoid of basic fibroblast growth factors. Then, the cells were allowed to spontaneously aggregate to form EBs. The differentiation medium, comprising DMEM–F12 (Invitrogen, Gibco) supplemented with 20% KnockOut Serum Replacement (Invitrogen, Gibco), 1 mM glutamine, 1% nonessential amino acids, 0.1% penicillin–streptomycin and 0.1 mM β-mercaptoethanol, was refreshed daily.

### Maintenance and reprogramming using the secondary reprogramming system

Human inducible fibroblast-like cells expressing ectopic human telomerase (hiF-T fibroblasts) for somatic cell reprogramming (from Davide Cacchiarelli; Broad Institute, Cambridge, MA, USA) were cultured in an optimized DMEM–F12 culture medium supplemented with 10% fetal bovine serum^27^. Reprogramming was performed using the TeSR-E7 Medium for Reprogramming (2-Component) containing Vitronectin XF and doxycycline hyclate (D9891, Sigma-Aldrich), according to the instructions of the manufacturer. Primosin (InvivoGen, ant-pm-1) at a concentration of 50 μg/ml was used to prevent microbial contamination during somatic cell reprogramming. hIF-T-derived iPS cells were cultured in TeSR-E8 medium (STEMCELL Technologies; 05990) or StemMACS iPS-Brew XF (Miltenyi Biotec, 130-104-368) containing Vitronectin XF.

### Optical microscopy

The morphologies of ES cells, iPS cell colonies and fibroblasts were observed using an optical microscope (CKX53; Olympus). Cell images were captured using an eXcope X9 device (Dixi Science).

### Electron microscopy

Cells were cultured on a 100-mm culture dish, washed twice with phosphate-buffered saline (PBS) and fixed with 2% paraformaldehyde–2.5% glutaraldehyde in 0.1 M phosphate buffer (pH 7.4) for 30 min. Then, the cell samples were lifted using a cell lifter (Corning), centrifuged (4000 rpm, 4 °C, 30 min) and stored at 4 °C until further processing. Next, the samples were postfixed in 1% osmium tetroxide, dehydrated and embedded in Eponate-12 resin (Ted Pella). One-micrometer-thick sections were prepared using a Reichert-Jung UltraCut E ultramicrotome (Reichert Technologies), stained with toluidine blue and imaged (Olympus BX-51). Seventy-nanometer-thick sections per block were placed on formvar-coated slot grids, stained with uranyl acetate–lead citrate and imaged using a transmission electron microscope (H-7600; Hitachi).

### Mito stress test

The oxygen consumption rate (OCR) and extracellular acidification rate (ECAR) were measured using a Seahorse XF96 extracellular flux analyzer (Seahorse Bioscience) according to the protocol of the manufacturer. Fibroblasts were plated in the wells of an XF96 cell culture microplate and incubated at 37 °C in a CO_2_ incubator for 24 h to ensure attachment. PS cells were plated onto the wells of an XF96 cell culture microplate coated with Vitronectin XF (STEMCELL Technologies, 07180) and incubated at 37 °C in a CO_2_ incubator for 24 h to ensure attachment. Ten micrometers of Y-27632 was added to the culture medium for the first 24 h after seeding. The assay was initiated after the cells were equilibrated for 1 h in XF assay medium supplemented with 10 mM glucose, 5 mM sodium pyruvate and 2 mM glutamine in a non-CO_2_ incubator. The substrate-based metabolic assay was performed by injecting 10 mM glucose after starvation in XF DMEM (pH 7.4; Seahorse Bioscience). The Mito stress test was carried out on the cells before and after sequential injection of 2 μM oligomycin, 1 μM carbonyl cyanide *m*‐chlorophenylhydrazone (CCCP), 0.5 μM rotenone and 0.5 μM antimycin A. Each OCR and ECAR value was normalized after protein quantification using the BCA Protein Assay Kit (Thermo Fisher Scientific, 23227) at the final step of the assay.

### RNA extraction, reverse transcription and quantitative real-time PCR

Cells were lysed with easy-BLUE (iNtRON Biotechnology), and total RNA was extracted. RNA was quantified using a NanoDrop ND-2000 spectrophotometer. One microgram of RNA was reverse-transcribed using the All-in-One 5× First Strand cDNA Synthesis Master Mix kit (CellScript), and quantitative PCR was performed using the TOPreal qPCR 2× PreMIX (Enzynomics). The experiments were performed according to the instructions of the manufacturer. RNA was extracted from cells under each treatment condition, and experiments were performed in triplicate. Primer sequences were designed using the Integrated DNA Technologies (IDT) online platform (https://sg.idtdna.com/pages). All primers were validated for efficiency.

### Western blotting

Cell lysates were extracted using NP-40 (Elpis Biotech) and a 100× protease/phosphatase inhibitor cocktail (Cell Signaling Technology, 5872S). Cell lysates were centrifuged at 13,000*g* for 20 min at 4 °C, and the supernatants were collected. The total protein concentration in each supernatant was determined using the BCA Protein Assay (Thermo Fisher Scientific, 23227). Then, the protein samples were separated by electrophoresis using 8–12% SDS–polyacrylamide gels and transferred to 0.2-μM polyvinylidene fluoride blotting membranes (Amersham). The membranes were blocked in 5% bovine serum albumin (BSA; BioShop, ALB001.100) in TBS-T (50 mM Tris, 0.15 M sodium chloride and 0.05% Tween 20) for 1 h at 25 °C. After blocking, the membranes were incubated with primary antibodies (1:1,000) diluted in 1% BSA in TBS-T for 16 h at 4 °C. Next, the membranes were washed three times with TBS-T for 10 min at room temperature and then incubated with horseradish-peroxidase-conjugated secondary antibodies (1:2,500) diluted in 1% BSA in TBS-T for 1 h at room temperature. Chemiluminescence was detected using a Pico EPD Western Blot Detection Kit (Elpis Biotech, EBP-1073) or the Amersham ECL Prime Western Blotting Detection Reagent (Amersham, RPN2232). The antibodies used in this study are listed in Supplementary Table 1.

### Meta-analysis of gene expression data

RNA sequencing (RNA-seq) data related to somatic cells, PS cells and reprogramming were collected and downloaded from the Gene Expression Omnibus (GEO) database. Subsequently, the STAR aligner software (STAR 2.7.10a, developed by Alexander Dobin, Cold Spring Harbor Laboratory) was used to map the data onto the GRCh38 reference genome. After mapping, the read counts obtained were used to analyze the expression levels of the genes of interest. Data (access codes) used for the analysis are provided in Supplementary Table 2. The results were visualized using R version 4.1.1 (The R Foundation for Statistical Computing). The ggplot2 function in R was used to create violin plots for visualization (https://ggplot2.tidyverse.org).

### Reverse-transcription quantitative PCR (RT-qPCR)

Cells were lysed using easy-BLUE (iNtRON Biotechnology), and total RNA was extracted. Then, RNA was quantified using a Nanodrop ND-2000 spectrometer (Thermo Fisher Scientific). Next, 1 μg of the RNA was reverse-transcribed using the cDNA Master Mix (CellSafe, CDS-100), and quantitative PCR was performed using the TOPreal qPCR 2X PreMIX (Enzynomics, RT500M). cDNA synthesis and qPCR were performed according to the instructions of the manufacturer. Primer sequences were designed using the IDT PCR primer design tool (https://sg.idtdna.com/pages). All primers were validated for efficiency and specificity, and experiments were performed in triplicate. Primer sequences are provided in Supplementary Table 3.

### mtDNA copy number analysis

A cell pellet was lysed using 500 μl of lysis buffer (Tris–HCl, 2 M pH 9.0; EDTA, 0.1 M pH 8.0; 0.5% SDS; and distilled water). Then, 10 μl of a 20 mg/ml proteinase K solution (Invitrogen; 25530049) was added, followed by incubation for 3 h at 55 °C or until complete dissolution of the cell pellet. Subsequently, following treatment with RNase A (Thermo Fisher Scientific; EN0531, 10 mg/ml) at 65 °C for 1 h, total cellular DNA was isolated using a conventional phenol–chloroform DNA extraction method. Then, the DNA was dissolved in Tris–EDTA (TE) buffer (10 mM Tris–HCl and 1 mM EDTA, pH 8.0); DNA from all samples was diluted to 50 ng. To determine mtDNA copy number, the nuclear DNA (nDNA)/mtDNA ratio was determined using the *ND1* gene primer specific to mtDNA and the genomic DNA β-globin gene primer. Primer sequences are provided in Supplementary Table 4. Quantitative PCR was performed using the TOPreal qPCR 2X PreMIX (RT500M, Enzynomics).

### ChIP and qPCR

Chromatin immunoprecipitation (ChIP) analysis was conducted using a TruChIP Chromatin Shearing Kit (Covaris, 520127). Initially, cells were subjected to treatment with methanol-free 16% paraformaldehyde (Thermo Fisher Scientific, 28908) for 3 min for fixation, followed by nuclear isolation. Subsequently, the isolated nuclei were fragmented to approximately 500 bp using a next-generation sequencing sample processor, M220 (Covaris). Immunoprecipitation was performed using specific antibodies and isotyped IgG, and antibody-bound chromatin was subsequently separated using Dynabeads Protein G (Invitrogen). Precipitated chromatin was treated with proteinase K (Invitrogen, 25530049, 20 mg/ml) and reverse cross-linked through heating at 65 °C for 4–5 h (or overnight). Then, after treatment with RNase A (Thermo Fisher Scientific, EN0531, 10 mg/ml) at 65 °C for 1 h, DNA was purified using a conventional phenol–chloroform DNA extraction method. Finally, target enrichment was quantitatively assessed using qPCR. Information regarding the primer sequences used is provided in Supplementary Table 5.

### Analysis of somatic cell reprogramming efficiency

Samples for reprogramming were dissociated into individual cells using the TrypLE Express Enzyme (1×) without phenol red (Gibco). After collecting the cells and washing them with PBS, single-cell suspensions were permeabilized and fixed in a 4% paraformaldehyde solution (in PBS). The cells were labeled with SSEA4 (APC) and TRA-1-60 (FITC) antibodies and then washed again. The fluorescence-activated cell sorting (FACS) antibodies used in this study are listed in Supplementary Table 1. All steps involving cell permeabilization, fixation, staining and data acquisition were performed on the same day using the same instrument for each experiment to maintain consistency. Analyses and cell sorting were performed using a FACS Canto II instrument from BD Biosciences, and data were processed using the BD FACSCanto Clinical Software (version 2.4, BD Biosciences).

### Statistical analysis

All experiments were performed using three independent biological replicates (*n* = 3), with each biological replicate containing three technical replicates (triplicate measurements per experiment). Data for statistical comparison are presented as mean ± standard deviation (s.d.). Statistical significance between groups was evaluated using Student’s *t*-test (two-tailed, unpaired). *P* values less than 0.05 were considered statistically significant. All statistical analyses were performed using the GraphPad Prism 8 software (GraphPad).

## Results

### Downregulation of *TK2* expression and mtDNA copy number is a molecular signature of human pluripotency

**Fig. 1:**
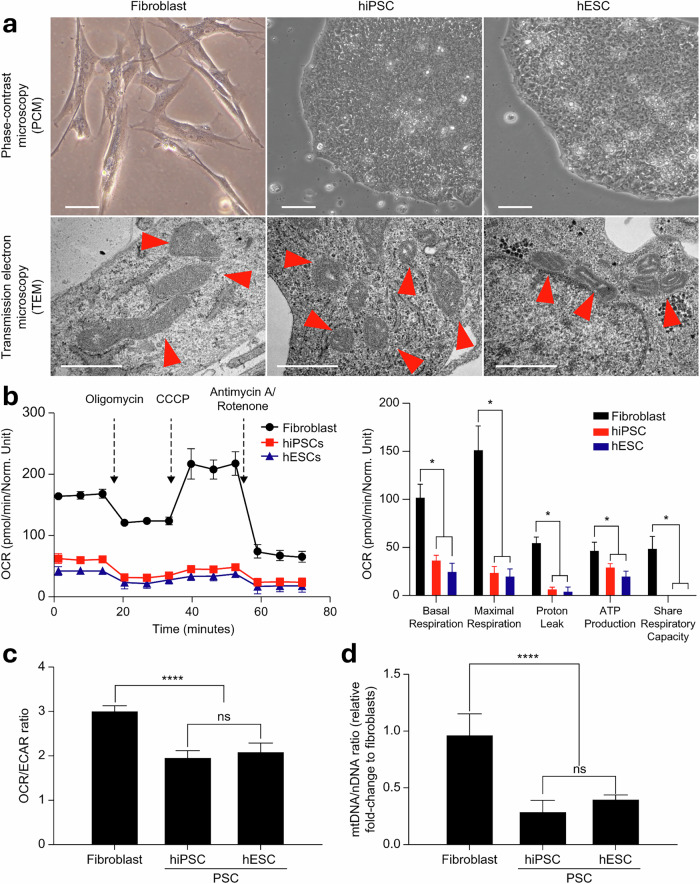
Mitochondrial function and morphological characterization in somatic cells and PSCs. **a** Phase-contrast microscopic (PCM) images depicting fibroblasts (MRC5; scale bar, 50 μm) and PSCs (hiPSC, H9-hESC; scale bar, 100 μm). Transmission electron microscopic (TEM) images of fibroblasts (MRC5) and PSCs (hiPSC, H9-hESC). Scale bar, 1 μm. Red arrows indicate mitochondria. **b** Mito stress test conducted on somatic cells and PSCs. Mito stress test data profiles for OCR and calculated values for respiratory parameters. Results are presented as means ± s.d. (*n* = 3). **c** Assessment of reliance on respiration or glycolysis in fibroblasts and PSCs as illustrated by the plotted OCR/ECAR ratios. Results are presented as means ± s.d. (*n* = 6). **d** mtDNA-to-nDNA ratios in fibroblasts and PSCs. Results are presented as means ± s.d. (*n* = 3). *P < 0.05, **P < 0.005, ***P < 0.0005, ****P < 0.00005). n.s., nonsignificant. Significant differences were assessed using Student’s *t*-test or one-way ANOVA with Tukey’s multiple-comparison test. Statistical analyses were performed using GRAPHPAD 8.0.1.ESC embryonic stem cell, PSC pluripotent stem cell.

To assess the metabolic changes that occur during somatic cell reprogramming, we compared mitochondrial morphological features between fibroblasts (MRC5) and PS cells (hiPS cells, CMC-003, and hES cells, H1) using electron microscopy. We found that fibroblasts contained mitochondria with dense cristae and elongated tubular shapes, while PS cells contained mitochondria with fewer cristae and round shapes (Fig. 1a). To determine whether these morphological differences between fibroblasts and PS cells corresponded with functional disparities, we evaluated mitochondrial function and the OCR/ECAR ratio using Seahorse XF96 and a series of drug injections consisting of an ATP synthase inhibitor (oligomycin), an uncoupler (CCCP) and electron transport chain inhibitors (antimycin A and rotenone). Human PS cells exhibited significantly lower overall OCRs and OCR/ECAR ratios than fibroblasts (*P* < 0.0001) (Fig. 1b, c). Specifically, during both basal and maximal respiration, the OCR in hPS cells was approximately two to five times lower than that in fibroblasts (*P* < 0.05) (Fig. 1b). In addition, OCR associated with mitochondrial inner membrane proton leakage was approximately 11-fold lower and ATP production-related OCR significantly decreased (*P* < 0.05) in hPS cells as compared with fibroblasts (Fig. 1b). Statistical analysis using a two-tailed Student’s *t*-test confirmed the significance of these metabolic differences, with 95% confidence intervals further supporting the observed decrease in mitochondrial respiration in hPS cells. Furthermore, energy mapping analyses demonstrated that fibroblasts maintained high OCRs, while reprogrammed cells exhibited a transition toward glycolytic metabolism (Fig. 1c, d). This result indicates that mitochondrial function and ATP production efficiency are relatively lower in hPS cells than in fibroblasts. Moreover, spare-respiratory capacity (calculated as maximum OCR minus basal OCR) was significantly lower in hPS cells than in fibroblasts (Fig. 1b), indicating that hPS cells maintain morphologically and functionally immature mitochondrial features. Of note, there was a marked difference in mtDNA copy number between fibroblasts and PS cells (Fig. 1d). mtDNA contains 13 genes critical for OXPHOS. Maintaining a certain mtDNA copy number is essential for mitochondrial function. Therefore, the metabolic shift that occurs during reprogramming is possibly due to changes in mtDNA copy number, which results in decreased mitochondrial function and acquired pluripotency.

### TK2 is a key regulator of mtDNA maintenance during reprogramming

**Fig. 2:**
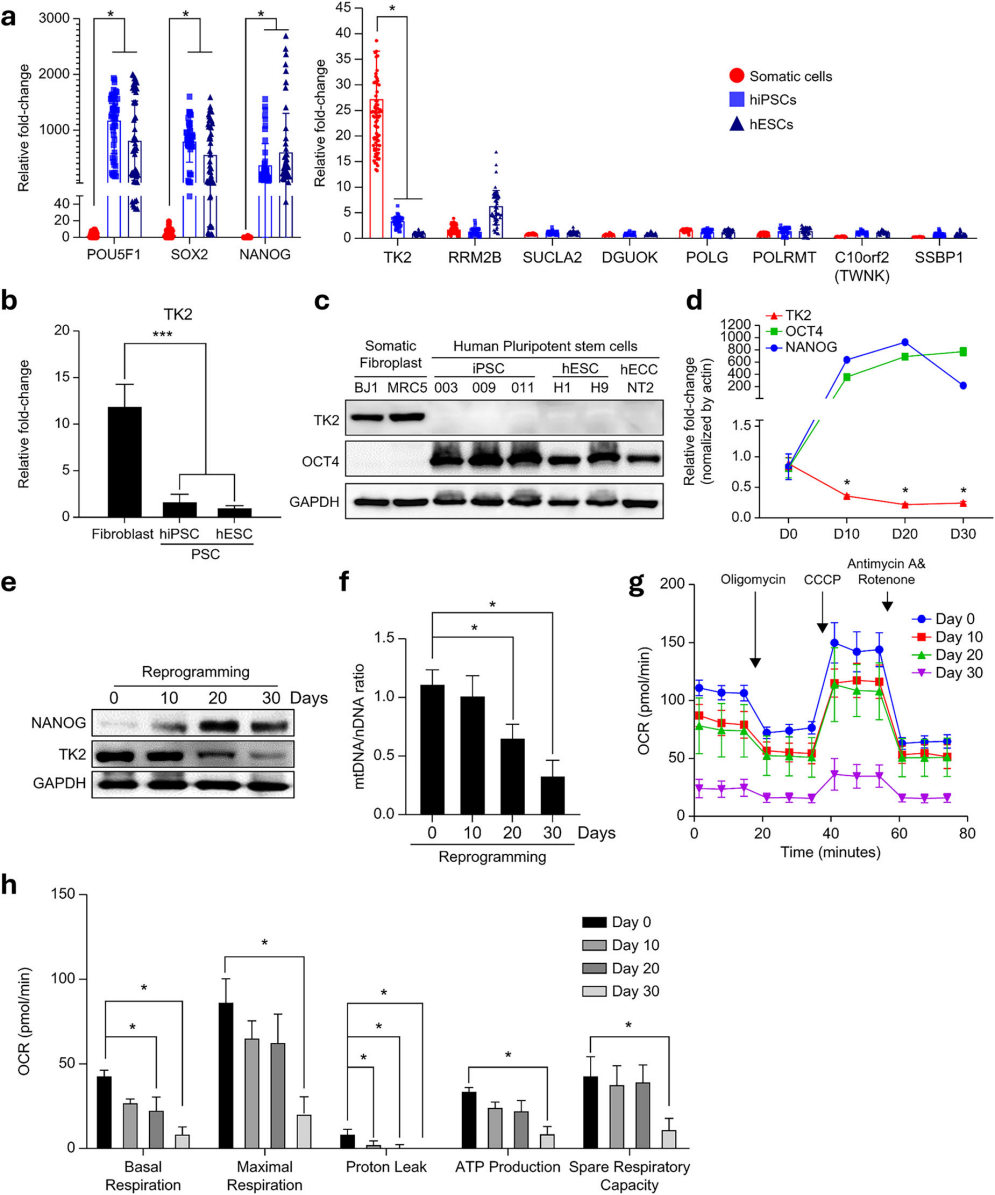
Decrease in *TK2* expression and mtDNA copy number as molecular signatures of human pluripotency. **a**, A violin plot illustrating log_2_-normalized read counts for mtDNA synthesis and replication-related genes in normal somatic cell lines (*n* = 78 and 29, respectively), hiPSC lines (*n* = 51) and hESC lines obtained through an RNA-seq database search. Results are presented as means ± s.d. **b**, RT-qPCR analysis depicting *TK2* expression in somatic cells and PSCs. **c**, Western blot analysis of TK2 protein expression in somatic cells and PSCs. **d**, RT-qPCR analysis of *TK2*, *OCT4* and *NANOG* expression patterns at days 10, 20 and 30 of the reprogramming process. **e**, Western blot analysis of TK2, NANOG and GAPDH protein expression patterns at days 10, 20 and 30 of reprogramming. **f**, Changes in the mtDNA/nDNA ratio during reprogramming. **g** Real-time measurement of the oxygen consumption rate (OCR) during mitochondrial stress test using the Seahorse XF analyzer. Each time point represents the average OCR of cells at day 0, 10, 20, and 30 during reprogramming. **h** Quantification of mitochondrial respiratory parameters derived from Seahorse assay shown in (g), including basal respiration, maximal respiration, proton leak, ATP production, and spare respiratory capacity. OCR values are compared across reprogramming time points (day 0, 10, 20, 30). Data represent mean ± SEM, with asterisks indicating statistically significant differences (*P < 0.05). **g**, **h** Results are presented as means ± s.d. (n = 3). *P < 0.05, **P < 0.005, ***P < 0.0005, ****P < 0.00005). Significant differences were assessed using one-way ANOVA and Tukey’smultiple-comparison test. Statistical analyses were performed using GRAPHPAD 8.0.1. ESC embryonicstem cell, PSC pluripotent stem cell.

To identify specific genes related to mtDNA replication and synthesis, we conducted a meta-analysis using open RNA-seq databases (Fig. 2a). RNA-seq data for somatic cells, iPS cells, ES cells and reprogramming processes were downloaded from the GEO database (Supplementary Table 2). After data mapping and normalization using a standardized pipeline, we analyzed the expression patterns of the target genes.

The constructed database accurately reflected the expression of pluripotency markers, such as OCT4, SOX2 and NANOG (Fig. 2a). Most genes involved in mtDNA synthesis and replication were significantly downregulated during reprogramming, with *TK2* exhibiting the most significant decrease (Fig. 2b and Supplementary Table 1). We speculate that, for mtDNA synthesis to occur efficiently and emerge as a pivotal factor in mitochondrial metabolic transition, thymidine supply, regulated primarily by TK2, takes precedence over other factors, underscoring the importance of TK2 in the regulation of mtDNA copy number. Thus, we sought to elucidate the functional characteristics of TK2 during reprogramming.

To validate the results obtained from the meta-analysis, we conducted RT-qPCR and western blot analyses on fibroblast cell lines and various PS cell lines (Fig. 2b, c). Consistent with the findings of the meta-analysis, we observed TK2 expression exclusively in somatic cell lines, with no expression observed in PS cell lines. Next, we used the human secondary reprogramming cell line, hiF-T, to evaluate the expression of genes related to mtDNA replication and synthesis. We evaluated genes related to mtDNA synthesis and replication using the mRNA sequencing data generated by Chicilla et al. during hiF-T cell reprogramming^27^. Of note, at the beginning of reprogramming, TK2 expression gradually decreased with increase in the degree of reprogramming (Supplementary Fig. 1). To validate these results, we evaluated both *TK2* mRNA and protein levels, the mtDNA/nDNA ratio and mitochondrial activity in hiF-T cells during somatic cell reprogramming (Fig. 2d–h). After doxycycline (DOX) treatment, fully reprogrammed hiF-T cells exhibited a decrease in both *TK2* mRNA and protein expression as compared with their baseline levels (day 30) (Fig. 2d, e).

Furthermore, as the reprogramming process advanced, the mtDNA/nDNA ratio significantly decreased, reaching a significance level 30% lower than that in somatic cells after a complete 30 days of reprogramming (Fig. 2f). Consistent with the decrease in TK2 expression and mtDNA copy number observed during reprogramming, a concurrent decrease in overall mitochondrial activity was observed (Fig. 2g, h). Basal respiration significantly decreased from day 20 of reprogramming, and a decreasing trend in maximal respiration, ATP production and spare respiration capacity was observed as reprogramming progressed. All mitochondrial metabolic parameters significantly decreased after 30 days of complete reprogramming. Of note, proton leakage markedly decreased after day 10 of reprogramming (Fig. 2h). These results suggest that, apart from the decrease in OXPHOS processes within mitochondria, there is also a decrease in the electrochemical gradient across the mitochondrial inner membrane during somatic cell reprogramming. This indicates that, as somatic cells undergo reprogramming to attain pluripotency, there is a concurrent decrease in both mtDNA copy number and mitochondrial function. To investigate whether TK2 expression increases in differentiating cells, we evaluated TK2 expression in spontaneously differentiated EBs derived from PS cells; we observed a time-dependent increase in TK2 expression in spontaneously differentiated EBs (Supplementary Fig. 2a, b). In addition, we observed distinct changes in TK2 expression between somatic cells and PS cells, indicating the need to investigate whether differences in TK2 expression can determine metabolic variations between these two cell types. In addition, as spontaneous EB differentiation progressed, the mtDNA/nDNA ratio increased. Specifically, the mtDNA/nDNA ratio in EBs that underwent differentiation for 15 days was over twofold higher than that in undifferentiated iPS cells (Supplementary Fig. 2c). Consistent with the trend in TK2 expression and the decrease in the mtDNA/nDNA ratio observed during spontaneous EB differentiation, a significant increase in specific mitochondrial parameters was observed (Supplementary Fig. 2d, e). In particular, there was a significant increase in maximal respiration, ATP production and spare respiration capacity on day 15 of spontaneous EB differentiation (Supplementary Fig. 2d, e). We observed distinct differences in TK2 expression between somatic cells and PS cells, underscoring the need to investigate whether differences in TK2 expression could determine metabolic variation.

### TK2 inhibition induces mitochondrial functional changes

**Fig. 3:**
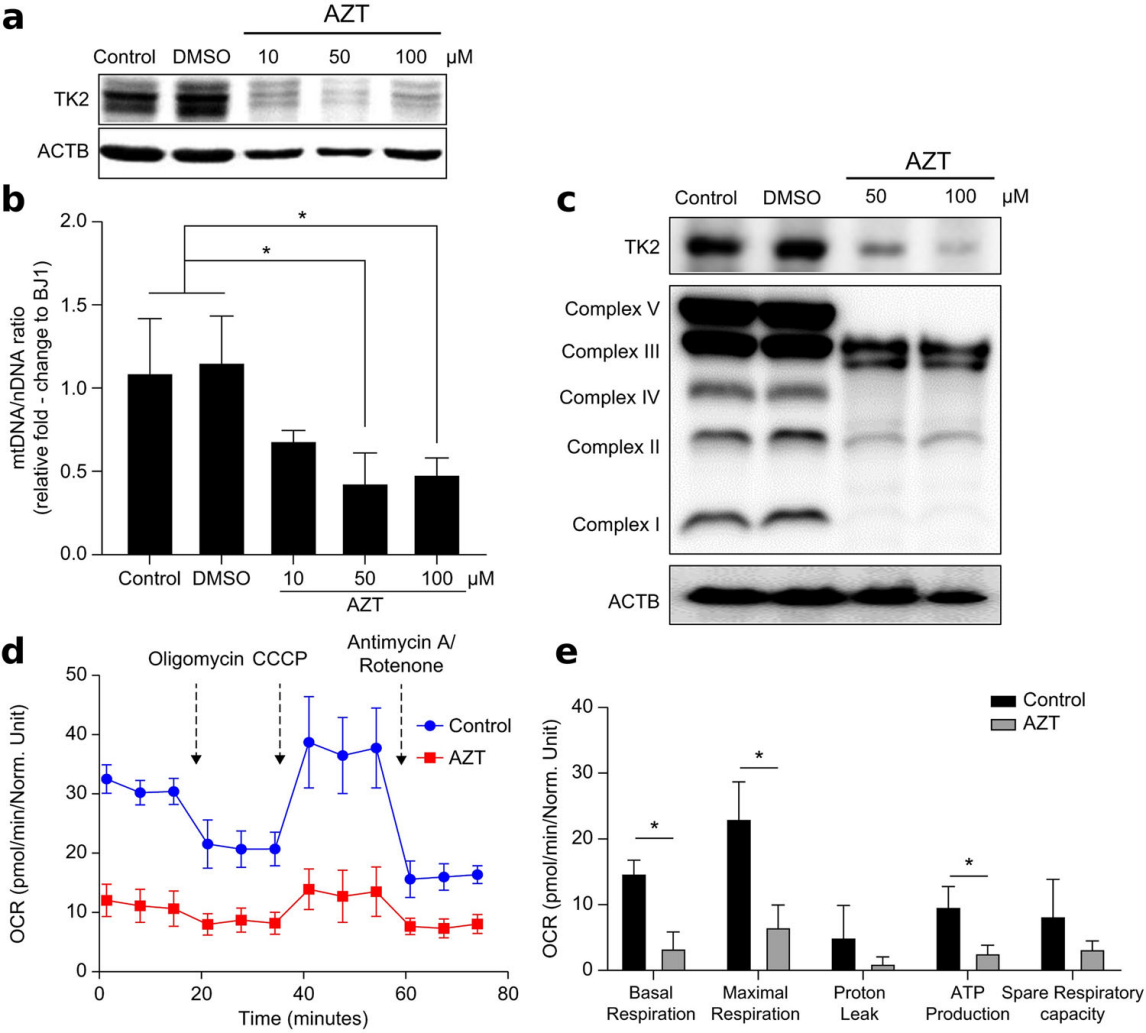
Inhibitory effect of TK2 in fibroblasts. **a** Western blot analysis of TK2 expression in fibroblasts (BJ1) after 72 h of treatment with AZT (a TK2 inhibitor). **b** Alterations in the mtDNA/nDNA ratio in fibroblasts (BJ1) after AZT treatment. Results are expressed as means ± s.d. (*n* = 3). **c** Changes in mitochondrial OXPHOS complex expression in fibroblasts (BJ1) after AZT treatment. **d** Real-time measurement of the oxygen consumption rate (OCR) during mitochondrial stress test in BJ1 fibroblasts treated with 50 μM AZT for 72 h. OCR was monitored at baseline and following sequential addition of oligomycin, CCCP, and a mixture of antimycin A and rotenone. **e** Quantitative analysis of mitochondrial respiration parameters derived from Seahorse assay shown in (**d**), including basal respiration, maximal respiration, proton leak, ATP production, and spare respiratory capacity. Results are presented as means ± s.d. (n = 4). Statistical significance was determined by Student’s t-test (*P < 0.05) **d**, **e**, Modulations in Mito stress test profiles and respiratory parameters in fibroblasts (BJ1) after 72 h of treatment with 50 μM AZT. Results are presented as means ± s.d. (n = 4). *P < 0.05. Significant differences were analyzed using Student’s t-test. Statistical analyses were conducted using GRAPHPAD 8.0.1. ESC, human embryonic stem cell, PSC pluripotent stem cell.

To determine whether TK2 expression directly regulates mitochondrial metabolic transition, we treated fibroblasts with TK2 inhibitors and assessed changes in mitochondrial function. BJ1 fibroblasts were cultured for 72 h with various concentrations of the TK2 inhibitor, 3′-azido-3′-deoxythymidin (AZT), to assess its impact on TK2 expression. TK2 protein expression decreased in a dose-dependent manner, and the mtDNA/nDNA ratio also significantly decreased (Fig. 3a, b). We evaluated the effects of AZT-induced mtDNA copy number reduction on protein expression in five OXPHOS complexes within the mitochondria. AZT reduced the expression of all five OXPHOS complexes, with no significant changes observed among them (Fig. 3c). Furthermore, to understand the effects of AZT treatment on cellular metabolic changes resulting from mtDNA copy number and OXPHOS complex reduction, we measured the OCR after AZT treatment (Fig. 3d, e). AZT treatment decreased overall OCR levels, as well as the values of five respiratory parameters. These results suggest that reducing mtDNA copy number through TK2 inhibition can lead to a transition in the functional characteristics of mitochondria from those of somatic cell mitochondria to those of PS cell mitochondria.

### Regulatory role of *p53* and SIRT1 in TK2 expression during reprogramming

**Fig. 4:**
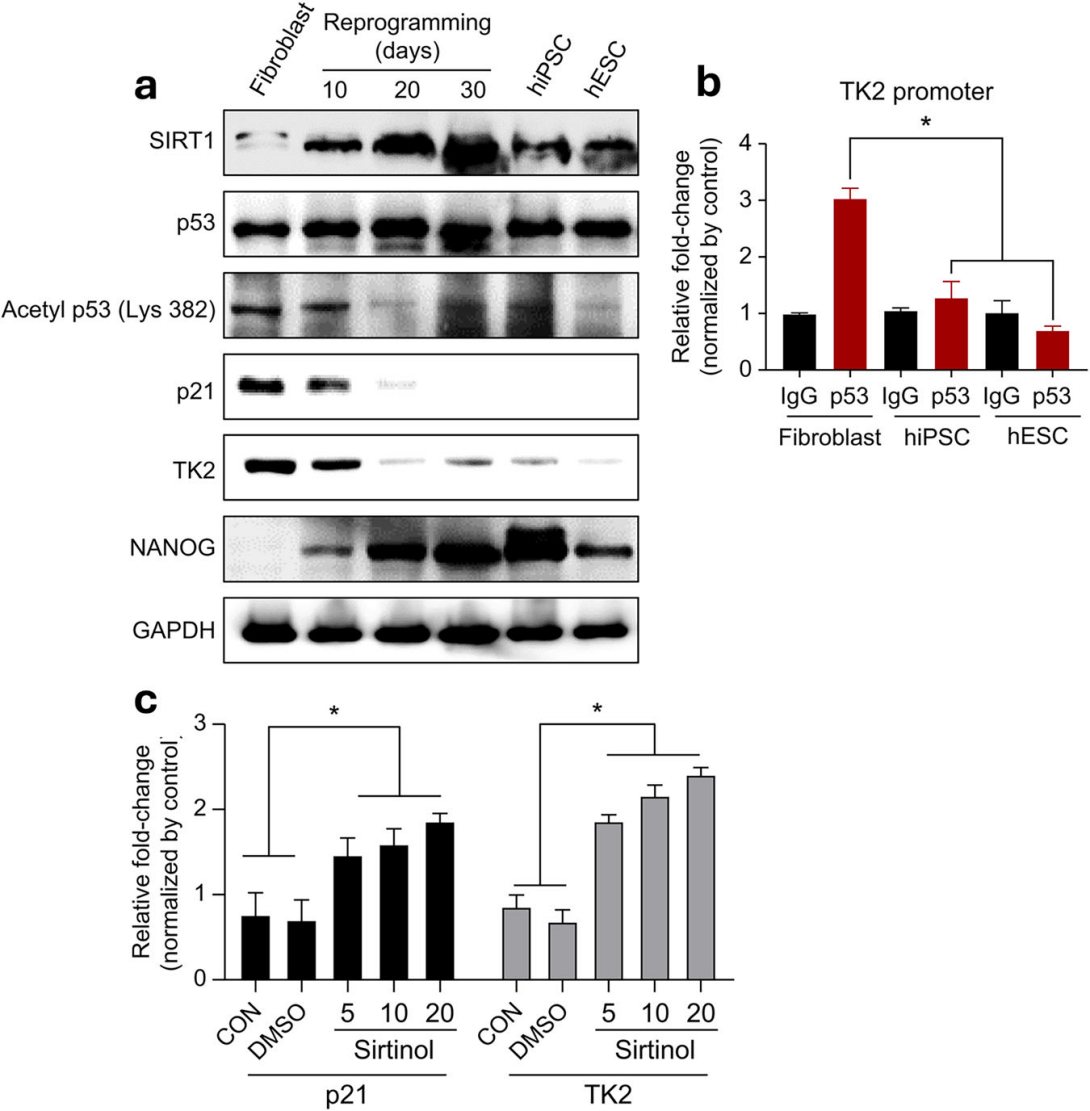
*p53* directly participates in TK2 transcription. **a**, Expression patterns of SIRT1, *p53* acetylation (Lys 382), p21, TK2 and NANOG during somatic cell reprogramming. **b**, ChIP-qPCR analysis illustrating the binding activity of *p53* to the TK2 promoter in fibroblasts and PSCs. **c** RT-qPCR analysis of p21 and TK2 expression in PSCs after 24 h of treatment with sirtinol (SIRT1 inhibitor). Results are presented as means ± s.d. (*n* = 3). *P < 0.05. Significant differences were analyzed using Student’s *t*-test or one-way ANOVA and Tukey’s multiple-comparison test. Statistical analyses were conducted using GRAPHPAD 8.0.1. ESC embryonic stem cell, PSC pluripotent stem cell.

The fact that decreased TK2 expression leads to reduced mtDNA copy number during the reprogramming process and contributes to a transition in cellular metabolism underscores the importance of exploring TK2 as a regulator of reprogramming. To identify potential upstream regulators of TK2 during the reprogramming process, we examined genes that could bind to the TK2 promoter using publicly available databases (ChIPBase v2.0)^28^, paying particular attention to *p53*. Not only is *p53* associated with cell cycle-related genes, it is also a well-known gene that can fine-tune PS cell stemness. Furthermore, we were particularly interested in *p53* as a potential upstream regulator of TK2 because its expression and mtDNA replication decrease in TP53-knockout and TP53-deficient subjects^26,29,30^. This suggests the existence of a relationship between reduced TK2 expression, decreased mtDNA replication and loss of *p53* function^22,31^. We first evaluated the expression of genes related to *p53* proteins in fibroblasts, PS cells and reprogrammed hiF-T cells using western blotting. A progressive increase in NANOG expression was observed in the reprogramming cell line, hiF-T, upon doxycycline treatment, indicating that reprogramming was indeed occurring (Fig. 4a). In contrast to NANOG expression, TK2 expression decreased in a time-dependent manner. Of note, while overall *p53* protein levels remained relatively constant across all cells, an acceleration in reprogramming was found to be associated with a decrease in *p53* acetylation (Lys 382). This decrease in *p53* acetylation was sustained at a low level even in iPS cell lines. In addition, decreased *p53* acetylation was correlated with a concurrent decrease in the expression of its downstream effector, p21. During reprogramming, the deacetylase, SIRT1, exhibited an expression pattern negatively correlated with *p53* acetylation levels (Fig. 4a). To determine whether *p53* directly regulates TK2 expression during the reprogramming process, we performed ChIP analyses and confirmed that *p53* indeed binds to the TK2 promoter. As compared with fibroblasts, PS cells exhibited a significant decrease in *p53* binding affinity toward both the p21 and TK2 promoter regions (Fig. 4b and Supplementary Fig. 3). Subsequently, to assess the impact of decreased SIRT1 expression on TK2 expression and *p53* activity, we treated cells with sirtinol, a SIRT1 inhibitor (Fig. 4c). Sirtinol treatment increased both TK2 and p21 expression levels. This increased p21 expression suggests that SIRT1 enhances *p53* activity, confirming that the SIRT1–*p53* axis is an upstream regulator of TK2 expression.

### SIRT1–TK2 axis-induced mtDNA depletion increases somatic cell reprogramming

**Fig. 5:**
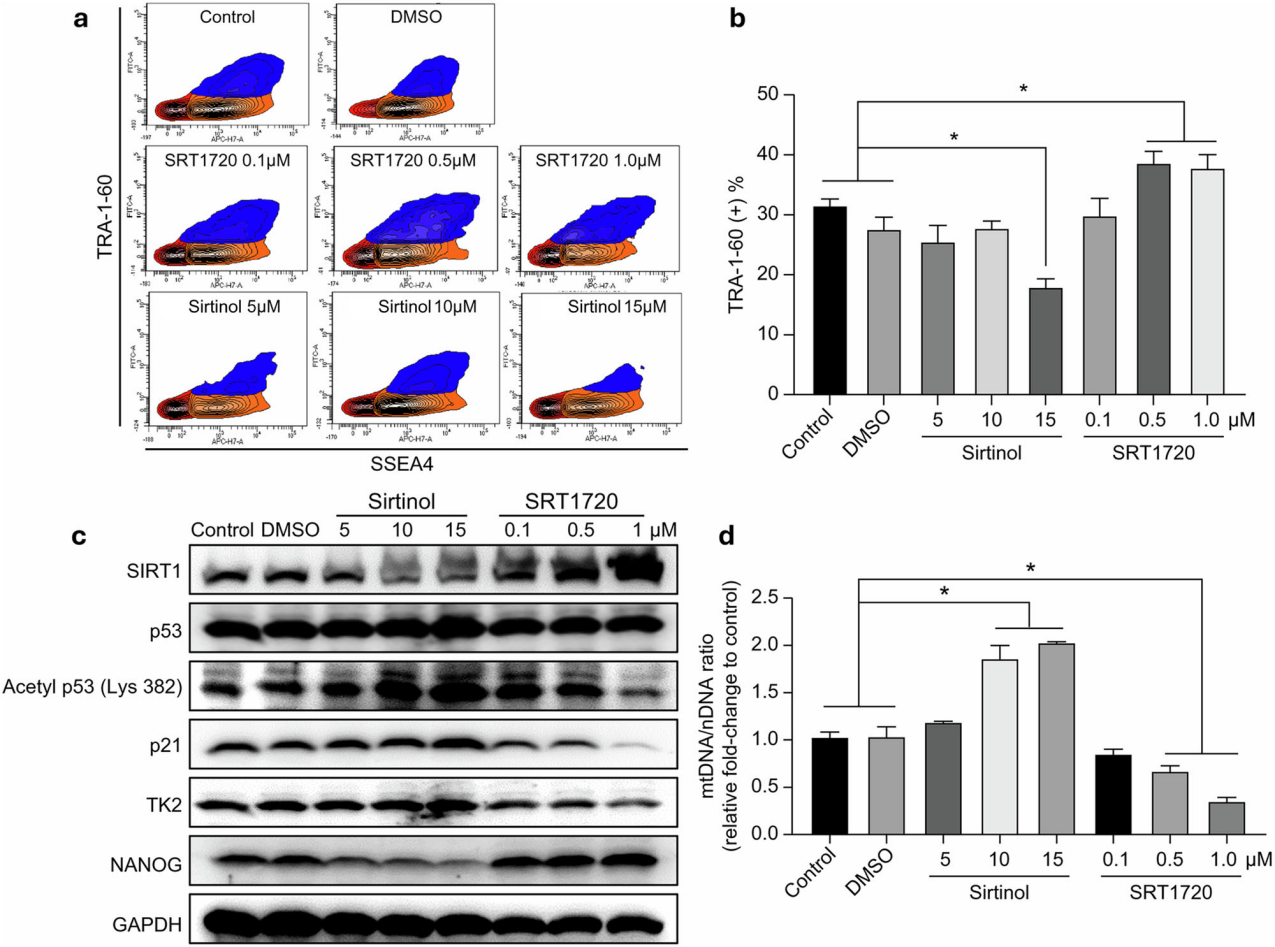
Assessment of somatic cell reprogramming efficiency using an SIRT1 inhibitor (sirtinol) and activator (SRT1720). **a**, Flow cytometric analysis of the surface markers, TRA-1-60 (FITC) and SSEA4 (APC), in sirtinol- and SRT1720-treated reprogrammed cells. **b**, Statistical analysis of somatic cell reprogramming efficiency based on FACS data. **c**, Alterations in the protein expression patterns of SIRT1, *p53* acetylation, p21, TK2 and NANOG due to SIRT1 regulation during somatic cell reprogramming. **d**, Changes in the mtDNA/nDNA ratio caused by SIRT1 regulation during somatic cell reprogramming. Results are presented as means ± s.d. (*n* = 3). The asterisk denotes statistical significance. Significant differences were assessed using Student’s *t*-test or one-way ANOVA and Tukey’s multiple-comparison test. Statistical analyses were conducted using GRAPHPAD 8.0.1.

We investigated whether SIRT1, an upstream regulator of TK2, directly modulates reprogramming through mtDNA depletion in hiF-T cells. To quantitatively assess the impact of SIRT1 regulation on reprogramming efficiency, we induced reprogramming in hiF-T cells through DOX treatment and conducted FACS analyses. Treatment with sirtinol at a concentration of 15 μM induced a decrease in the SSEA4–TRA-1-60 double-positive cell population by approximately 30% (Fig. 5a, b), whereas treatment with SRT1720 during hiF-T cell reprogramming resulted in an approximate 27% increase in reprogramming efficiency (Fig. 5a, b). Treatment with sirtinol increased *p53* and TK2 expression and decreased NANOG expression in hiF-T cells that underwent reprogramming for 20 days. Conversely, treatment with the SIRT1 activator, SRT1720, decreased *p53* and TK2 levels and increased NANOG expression (Fig. 5c). SIRT1 activity was positively correlated with mtDNA expression (Fig. 5d). This indicates that the SIRT1–*p53* axis can directly regulate metabolic transition in reprogrammed cells, enhancing reprogramming efficiency.

## Discussion

This study revealed that a decrease in mtDNA copy number, which is crucial for the transition from OXPHOS-driven metabolism to aerobic glycolysis, is necessary for somatic cells to acquire and maintain pluripotency while undergoing stochastic processes, with a key factor being the downregulation of *TK2*, which is involved in mitochondrial thymidine biosynthesis. The TK enzyme, responsible for converting thymidine into its monophosphate form (dTMP; essential for DNA synthesis and repair), was first identified in the 1960s as an enzyme that adds a 5′ phosphate to thymidine-deoxyribose^32^. Subsequently, two isoforms of TK, TK1 and TK2, were identified. TK1, located on chromosome 17 (17q25.3), is highly proliferative and plays a role in nDNA synthesis, particularly in rapidly dividing fetal and tumor tissues^32^. By contrast, TK2, located on chromosome 16 (16q21), is responsible for mtDNA replication and is not involved in cell cycle regulation^32^. In this study, we highlight the metabolic shift from OXPHOS to aerobic glycolysis during somatic cell reprogramming, emphasizing its parallels and distinctions with the canonical Warburg effect observed in cancer cells. While both PS cells and cancer cells rely on glycolysis, the purpose of this process differs significantly between the two cell types: PS cells adopt this metabolic reprogramming to support the biosynthetic and energy demands of pluripotency acquisition, driven by Yamanaka factors, whereas cancer cells utilize the Warburg effect for uncontrolled proliferation under hypoxic conditions, mediated by oncogenic drivers such as HIF-1α and c-Myc. TK2 plays a distinct role in both contexts; its suppression in PS cells facilitates this metabolic shift by reducing mtDNA replication and mitochondrial activity, enabling a glycolytic phenotype essential for reprogramming. Conversely, in cancer cells, TK2 maintains mtDNA stability and regulates reactive oxygen species, promoting metabolic flexibility and tumorigenic survival. These findings underscore the unique role of TK2 in metabolic dynamics across different biological systems, providing deeper insights into its regulatory mechanisms during reprogramming. Thus, while PS cells and cancer cells share metabolic features such as dependence on glycolysis and altered mitochondrial metabolism, they significantly differ in their regulation of TK2 and TK1. In cancer cells, rapid proliferation leads to abnormal TK1 activation, reducing reliance on TK2, whereas PS cells exhibit selective TK2 downregulation as part of their metabolic transition toward glycolysis and reduced mitochondrial activity. Therefore, TK2 downregulation is a metabolic hallmark of pluripotency, whereas its maintenance in cancer cells is a consequence of TK1-driven nucleotide metabolism rather than a defining metabolic adaptation.

We observed that TK1 transcription and translated protein levels remained constant, while TK2 levels changed (Supplementary Fig. 4a,b,d). We observed that TK1 transcription and translated protein levels remained constant across pluripotent cells, reprogrammed cells and fibroblasts, while TK2 levels changed depending on the cell state, with reduced expression in pluripotent and reprogrammed cells compared with fibroblasts (Supplementary Fig. 4a,b,d). To further investigate TK1 and TK2 protein synthesis on the basis of cellular characteristics, we treated fibroblasts with nocodazole to inhibit the cell cycle and then analyzed their protein expression (Supplementary Fig. 4c). Our analyses showed that cell cycle inhibition in fibroblasts (BJ1) significantly decreased TK1 protein expression but did not affect TK2 expression. Based on these data, the lower expression of TK1 in somatic cells as compared with PS cells can be attributed, in part, to relative differences in the cell cycle between these two cell types. However, unlike TK1, TK2 is expressed independently of the cell cycle, indicating its involvement in mtDNA synthesis regardless of the cell cycle stage. While TK2 expression is cell cycle independent, its low expression in PS cells, despite their vigorous cell cycle activity, is probably due to a metabolic shift toward pluripotency maintenance and a preference for nucleic acid synthesis through the pentose phosphate pathway, which is associated with metabolic transition towards aerobic glycolysis.

Our findings underscored the significance of decreased TK2 expression metabolic transition regulation during the reprogramming process. In addition, through multiple experimental approaches, we identified TK2 as a crucial regulator of mtDNA synthesis and replication. Our meta-analysis of transcriptomic datasets (Fig. 2) revealed that, compared with the expression of other mtDNA synthesis-related genes, such as *POLG* and *TFAM*, *TK2* expression decreases more significantly during somatic cell reprogramming, particularly during the early-to-intermediate stages. This suggests that TK2 is closely associated with metabolic transitions during reprogramming. Functionally, TK2 is essential for salvaging mitochondrial thymidine, thereby maintaining the nucleotide pool required for mtDNA replication. Unlike direct promoters of pluripotency, TK2 primarily regulates metabolic remodeling, which is critical for the success of reprogramming. Aside from its role in reprogramming, we explored the broader biological significance of TK2 by analyzing its expression across various human tissues. Using publicly available datasets, we found a significant positive correlation between TK2 expression and mtDNA copy number (Pearson *r* = 0.481, *P* = 0.0319) (Supplementary Fig. 5). Of note, metabolically active tissues such as skeletal muscle, heart tissue and tissues of specific brain regions (cortex, hippocampus and basal ganglia) exhibit the highest TK2 expression levels, which is consistent with their high mitochondrial demands. Conversely, tissues with lower metabolic activity, such as splenic and small intestinal tissue, exhibit decreased TK2 expression and mtDNA copy numbers. These findings suggest that TK2 plays a fundamental role in mitochondrial homeostasis beyond PS cells, potentially regulating tissue-specific energy metabolism and mitochondrial maintenance. In addition, we conducted mtDNA depletion and TK2 inhibition experiments using ethidium bromide (EtBr) and AZT. If mtDNA depletion directly enhanced pluripotency, further reduction of its expression would have increased reprogramming efficiency. However, excessive mtDNA loss induced by EtBr treatment impaired reprogramming, indicating that severe metabolic stress disrupts the process. Similarly, AZT treatment reduced reprogramming efficiency, suggesting that TK2 inhibition dysregulates the mitochondrial nucleotide balance and metabolic homeostasis (Supplementary Fig. 6). These findings collectively indicate the role of TK2 in coordinating metabolic adaptation rather than merely reducing mtDNA levels, highlighting its distinct importance in this process. Furthermore, to better understand stochastic reprogramming, we sought to identify upstream regulators of TK2 and found that a critical regulatory axis, consisting of Oct4, SIRT1 and *p53*, is involved in mtDNA copy number modulation during somatic cell reprogramming. Oct4-induced SIRT1 expression leads to *p53* deacetylation and inhibition, thereby disrupting its positive regulation of *TK2*. This suppression leads to decreased TK2 expression, lowering thymidine availability and contributing to the observed decrease in mtDNA copy number, and this facilitates the metabolic shift required for pluripotency. Previous studies have shown that *p53* directly binds to the TK2 promoter (ChIPbase2.0) and positively regulates its transcription, while the inhibitory effect of SIRT1 on *p53* during reprogramming has been demonstrated^33,34^ These findings underscore the role of SIRT1 as a central integrator of nuclear and mitochondrial gene expression, highlighting its influence on mtDNA dynamics during metabolic remodeling in PS cells. Further studies could validate this pathway through the exploration of SIRT1 and *p53* activity, as well as the assessment of their direct effects on TK2 expression and mtDNA regulation.

Through the analysis of publicly available literature, we confirmed that *p53* directly binds to the TK2 promoter, thereby exerting its regulatory effects. The importance of *p53* expression and acetylation in reprogramming and maintenance of pluripotency has been well established^33–36^. SIRT1 is a member of an extensive family of histone deacetylases, and it regulates *p53* activity, influences the fate of numerous cells and maintains homeostasis. One reason for the extremely low reprogramming efficiency in cells is that all reprogramming factors associated with the cell cycle, particularly c-Myc and klf4, are potent oncogenic proteins, leading to the potential activation of *p53* during reprogramming^37^. Activated *p53* halts the cell cycle, induces cell death and promotes aging, thereby inhibiting cell reprogramming. Consequently, regulating *p53* activity during reprogramming is a major bottleneck to reprogramming efforts. As SIRT1 can regulate *p53* activity from an upstream position, it plays a key role in maintaining genomic stability and inducing metabolic transition during reprogramming. Specifically, SIRT1 is directly transcribed by OCT4; therefore, ectopic OCT4 expression increases SIRT1 expression, thereby reducing *p53* acetylation. It is speculated that the decrease in mtDNA copy number and TK2 expression during somatic cell reprogramming is a consequence of OCT4 activity^33,34^.

TK2 inhibition was initially expected to improve reprogramming efficiency as it has been reported to induce the Warburg effect and mitochondrial function. As shown in Fig. 3, we found that OXPHOS complexes exhibited differential sensitivity to TK2 inhibition during somatic cell reprogramming, as indicated by the consistent decrease in protein expression across all five complexes after AZT treatment. Complexes I, III, IV and V, which are partly encoded by mtDNA, exhibited decreased expression levels in line with the global decrease in mtDNA copy number under TK2 inhibition. Interestingly, complex II, which is entirely nuclear-encoded, also exhibited decreased expression. This unexpected finding probably reflects the indirect effects of mitochondrial dysfunction, such as disrupted coordination between nuclear and mitochondrial gene expression or altered signaling pathways. In a previous study^38^, we demonstrated that miR-31 induces these metabolic changes by repressing the expression of SDHA, a key component of mitochondrial electron transport chain complex II. Building on this, we demonstrate here that TK2-mediated changes in mtDNA replication selectively influence complexes I, III, IV and V, offering new insights into mitochondrial remodeling during reprogramming. This variability in the magnitude of decrease in the expression of the complexes possibly indicates that they exhibit different levels of dependency on mtDNA-encoded subunits. Complex I is highly dependent on mtDNA, whereas complex II may be indirectly affected by mitochondrial stress despite being nuclear encoded. These findings provide deeper insights into OXPHOS vulnerability to TK2 inhibition and its pivotal role in driving the metabolic adaptations essential for pluripotency.

Directly blocking TK2 expression exerts potential adverse effects on somatic cell reprogramming processes. TK2 mutant mice (TK2(KI/KI)) and conditions associated with mtDNA depletion are associated with high AMPK activity^28^. Moreover, AMPK acts as a metabolic checkpoint, thereby inhibiting somatic cell reprogramming^29^. During OSKM-induced somatic cell reprogramming, decreased mitochondrial function leads to a decrease in ATP levels, which may trigger an AMPK-mediated energy homeostatic response. This suggests that energy metabolism and homeostatic mechanisms negatively affect pluripotency acquisition during cell reprogramming. Consequently, we observed that treatment with a TK2 inhibitor impaired somatic cell reprogramming, underscoring the role of TK2 in regulating mitochondrial metabolism and mtDNA dynamics. TK2 suppression leads to a decrease in mtDNA copy number, and this disrupts mitochondrial function and ATP production, resulting in metabolic stress and the activation of the AMPK–*p53* signaling axis. This axis serves as a metabolic checkpoint, inhibiting dedifferentiation and decreasing reprogramming efficiency. While TK2 downregulation is associated with metabolic transition from OXPHOS to glycolysis, its inhibition exacerbates energy imbalance and reactive oxygen species production, creating a cellular environment that impairs reprogramming. These findings suggest that TK2 not only reflects metabolic remodeling but also actively contributes to the regulation of mitochondrial homeostasis, highlighting its pivotal role in balancing energy metabolism during pluripotency acquisition. This suggests that a total absence of TK2 may induce metabolic stress, DNA damage and replication stress during the reprogramming process, potentially negatively affecting pluripotency acquisition. The primary challenge with investigating alterations in reprogramming efficacy using AZT lies in the possible adverse impact of direct TK2 expression inhibition on somatic cell reprogramming. Given the intricate nature of cellular mechanisms, AZT administration may lead to unforeseen repercussions or off-target effects associated with TK2 suppression, potentially influencing overarching outcomes. However, iPS cells can also be successfully established using TK2-mutated fibroblasts^39^. Of note, these results are predominantly obtained in cells that underwent complete metabolic adaptation following TK2 knockout. Therefore, direct TK2 expression inhibition should be carefully fine-tuned during somatic cell reprogramming.

## Supplementary information

Supplementary Information

## Data Availability

The Supplementary Information for this Article is provided on the *Experimental & Molecular Medicine* website (http://www.nature.com/emm/).
