# Author Correction: Changes in mitochondrial thymidine metabolism and mtDNA copy number during induced pluripotency

**DOI:** 10.1038/s12276-025-01617-8

**Published:** 2026-01-19

**Authors:** Hyun Kyu Kim, Yena Song, Minji Kye, Byeongho Yu, Hyung Kyu Choi, Sung-Hwan Moon, Man Ryul Lee

**Affiliations:** 1 Soonchunhyang Institute of Medi-bio Science, Soon Chun Hyang University, Cheonan, Republic of Korea; 2 Dementia Research Group, Korea Brain Research Institute, Daegu, South Korea; 3 Department of Animal Science and Technology, Chung-Ang University, Anseong, Republic of Korea; 4 Department of Stem Cell and Regenerative Biotechnology, KU Institute of Science and Technology, The Institute of Advanced Regenerative Science, Konkuk University, Gwangjin-gu, Republic of Korea

**Keywords:** Induced pluripotent stem cells, Reprogramming

Correction to: *Experimental & Molecular Medicine* 10.1038/s12276-025-01476-3, published online 26 June 2025

After online publication of this article, the authors noticed an error in the Fig. 5a section.

In Fig 5a; SRT1720 5 μM, SRT1720 10 μM, SRT1720 15 μM should have appeared Sirtinol 5 μM, Sirtinol 10 μM, Sirtinol 15 μM. The manuscript text remains unchanged; only the labels in Fig. 5a require correction.

Original Figure 5:
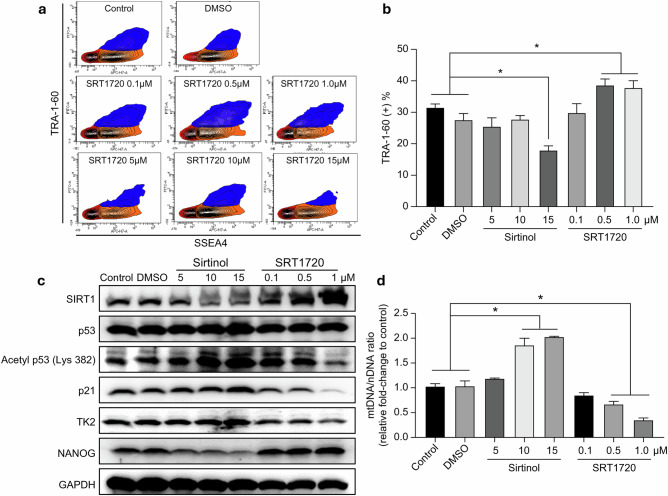


Corrected Figure 5:
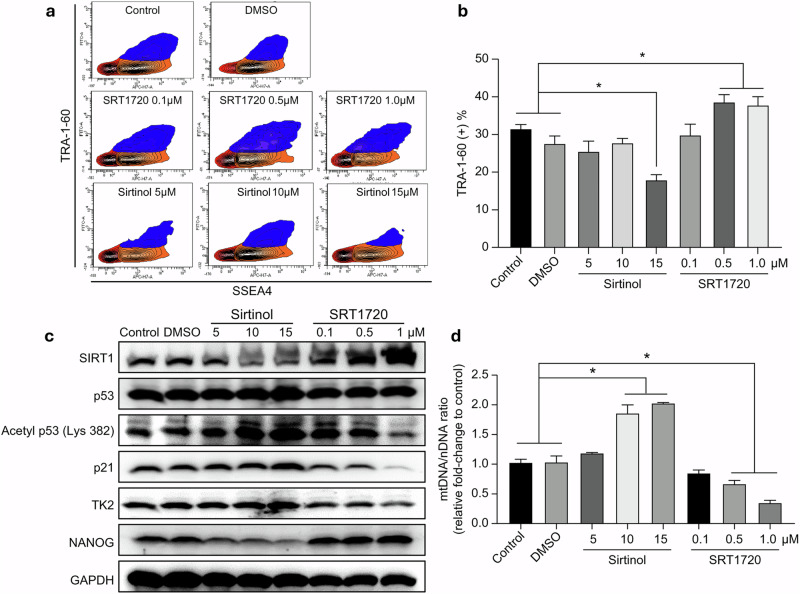


The original article has been corrected.

