# Supplementary material for: Changes in mitochondrial thymidine metabolism and mtDNA copy number during induced pluripotency
Source: Exp Mol Med. 2025 Jun 26;57(6):1272–82. doi: 10.1038/s12276-025-01476-3 (PMC12229447; doi:10.1038/s12276-025-01476-3)
Supplement: Supplementary file 1 — Supplementary Information [file 12276_2025_1476_MOESM1_ESM.pdf]

Supplementary Materials

Changes in mitochondrial thymidine metabolism and mtDNA copy number during induced pluripotency

Hyun Kyu Kim[1, 2], Yena Song [1], Minji Kye[1], Byeongho Yu [1], Hyung Kyu Choi[3], Sung-Hwan Moon[3] and Man Ryul Lee[4,*]

1. Soonchunhyang Institute of Medi-bio Science (SIMS), Soon Chun Hyang University, Cheonan, 31151, Republic of Korea
2. Dementia Research Group, Korea Brain Research Institute (KBRI), Daegu, South Korea
3. Department of Animal Science and Technology, Chung-Ang University, Anseong, Gyeonggi-do, 17546, Republic of Korea
4. Department of Stem Cell and Regenerative Biotechnology, KU Institute of Science and Technology, Konkuk University, 120 Neungdong-ro, Gwangjin-gu, Seoul 05029, Republic of Korea

This file contains:

Supplementary Tables 1 to 5

Supplementary Figures 1 to 6

Supplementary Table 1. List of antibodies used in the experiment.

Name	Host or isotype	Manufacturer	Cat No.	Application
Acetyl-p53 (Lys382)	Rabbit	Invitrogen	710294	WB
GAPDH	Mouse	Santa Cruz Biotechnology	sc-47724	WB
Goat anti-mouse IgG - HRP	Goat	Santa Cruz Biotechnology	sc-2031	WB
Mouse anti-rabbit IgG - HRP	Mouse	Santa Cruz Biotechnology	sc-2357	WB
NANOG	Rabbit	Cell signaling technology	4903S	WB
OCT4	Mouse	Santa Cruz Biotechnology	sc-5279	WB
Normal Mouse IgG	Mouse	MerckMillipore	12-371	ChIP
p21	Mouse	Santa Cruz Biotechnology	sc-6246	WB
p53	Mouse	Santa Cruz Biotechnology	sc-126	WB
p53	Mouse	MerckMillipore	CS200578	WB, ChIP
SIRT1	Mouse	Santa Cruz Biotechnology	sc-74465	WB
TK1	Rabbit	Cell signaling technology	8960	WB
TK2	Rabbit	ATLAS	HPA041162	WB

		ANTIBODIES		
Total OXPHOS complexes	Mouse	Abcam	ab110413	WB
SSEA4 - Alexa Fluor® 647	Mouse	BD Biosciences	560796	FACS
TRA-1-60 - FITC	Mouse	BD Biosciences	560876	FACS

ChIP, chromatin immunoprecipitation; FACS, fluorescence-activated cell sorting; WB, western blotting.

Supplementary Table 2. Transcriptome data used for the meta-analysis.

Cell type	Data	Cell type	Data	Cell type	Data	Cell type	Data
	SRR1660534		SRR1633330		SRR1268174		SRR1268171
	SRR1660535		SRR1633331		SRR1268175		SRR2038463
	SRR1660536		SRR1633332		SRR1268176		SRR2038464
	SRR1660537		SRR1633333		SRR1268177		SRR2038465
	SRR1660538		SRR1633334		SRR1633349		SRR2038466
	SRR1660539		SRR6762314		SRR1633350		SRR2038467
	SRR1660540		SRR6762315		SRR1633351		SRR2038468
	SRR1660541		SRR6762316		SRR1633352		SRR2038469
	SRR1660542		SRR1633303		SRR1633353		SRR2038470
	SRR1660543		SRR1633304		SRR1633354		SRR2038471
	SRR1660544		SRR1633305		SRR1633355		SRR2038472
	SRR1660545		SRR1633306		SRR1633356		SRR2038473
**Somatic cells**	SRR1660546	**Somatic cells**	SRR1633307	**hiPSC**	SRR1633357	**hESC**	SRR2038474
	SRR1660547		SRR1633308		SRR1633358		SRR2038475
	SRR1660548		SRR1633309		SRR1633359		SRR2038476
	SRR1660549		SRR1633310		SRR1633360		SRR2038477
	SRR1660550		SRR1633311		SRR1633361		SRR2038478
	SRR1660551		SRR1633312		SRR1633362		SRR2038479
	SRR1660552		SRR1633313		SRR1633363		SRR2038480
	SRR1660553		SRR1633314		SRR1633364		SRR2057152
	SRR1660554		SRR1633315		SRR2038481		SRR2057156
	SRR1660555		SRR1633316		SRR2038482		SRR2057164
	SRR1660556		SRR1633317		SRR2038483		SRR2057167
	SRR1660557		SRR1633318		SRR2038484		SRR2057169
	SRR1660558		SRR1633319		SRR2038485		SRR2057185

	SRR1660559		SRR1633320		SRR2038486		SRR2057189
	SRR1660560		SRR1633321		SRR2038487		
	SRR2751110		SRR1633322		SRR2038488		
	SRR2751111		SRR1633323		SRR2038489		
	SRR2751112		SRR1633324		SRR2038490		
	SRR2751113		SRR1633325		SRR2038491		
	SRR2751114		SRR1633326		SRR2038492		
	SRR2751115		SRR1633327		SRR2057132		
	SRR2751116		SRR1633328		SRR2057137		
	SRR2751117		SRR1044666		SRR2057142		
	SRR2751118		SRR1044667		SRR2057147		
	SRR2751120		SRR1044668		SRR2057176		
	SRR2751121		SRR1044669		SRR2057181		
	SRR2751122	**hiPSC**	SRR1044672		SRR2057209		
	SRR2751123		SRR1044673		SRR2057214		
	SRR2751124		SRR1044674		SRR2057219		
	SRR2751125		SRR1044675		SRR1044670		
	SRR2751126		SRR1268172	**hESC**	SRR1044671		
	SRR2751127		SRR1268173		SRR1268170		

hESC, human embryonic stem cell; hiPSC, human induced pluripotent stem cell.

Supplementary Table 3. List of primers used in the RT-qPCR analysis[a].

Gene	Sequence (5'→3')
*ACTB*_F	CAT GTA CGT TGC TAT CCA GGC
*ACTB*_R	CTC CTT AAT GTC ACG CAC GAT
*GAPDH*_F	GGT GTG AAC CAT GAG AAG TAT GA
*GAPDH*_R	GAG TCC TTC CAC GAT ACC AAA G
*CDKN1A*_F	CCT GTC ACT GTC TTG TAC CCT
*CDKN1A*_R	GCG TTT GGA GTG GTA GAA ATC
*NANOG*_F	CTC CCT AAC AGC TGG GAT TTA
*NANOG*_R	GAC GGC AGC CAA GGT TAT TA
*OCT4*_F	GGA GGA AGC TGA CAA CAA TGA
*OCT4*_R	CTC TCA CTC GGT TCT CGA TAC T
*SOX2*_F	GAG AGA AAG AAG AGG AGA GAG AAA G
*SOX2*_R	GCC GCC GAT GAT TGT TAT TAT T
*TK1*_F	CAC AGG AAC AAC AGC ATC TTT C
*TK1*_R	CCT CTC CAC ACT TGA AGA GAT AAG
*TK2*_F	CAT CTG TAC GGT TGA TGG AGA G
*TK2*_R	CAT AGT CCA CTT CTG GCA TCT T

[a] RT-qPCR, reverse transcription quantitative polymerase chain reaction.

Supplementary Table 4. List of primers used in the mtDNA[a] copy number analysis.

Gene	Sequence (5'→3')
ND1-F	CCC TAA AAC CCG CCA CAT CT
ND1-R	GAG CGA TGG TGA GAG CTA AGG T
β-globin-F	CTA TGG GAC GCT TGA TGT
β-globin-R	GCA ATC ATT CGT CTG TTT

[a] mtDNA, mitochondrial DNA.

Supplementary Table 5. List of primers used in the ChIP-qPCR analysis.

Gene	Sequence (5'→3')
CDKN1A promoter F	CCC ACA GCA GAG GAG AAA GAA
CDKN1A promoter R	CTG GAA ATC TCT GCC CAG ACA
TK2 promoter F	TCA TCT CAG CAA GAG GAG AAA TAC
TK2 promoter R	CTC CGA GTG TTG CCA AGT TA

[a] ChIP-qPCR, chromatin immunoprecipitation-quantitative polymerase chain reaction.

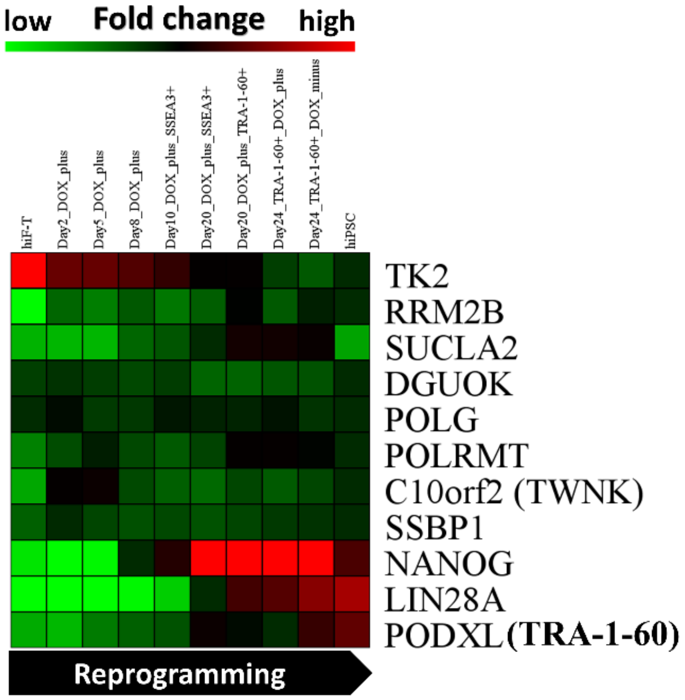

Supplementary Fig. 1

Heatmap of mtDNA replication- and synthesis-related genes during reprogramming.

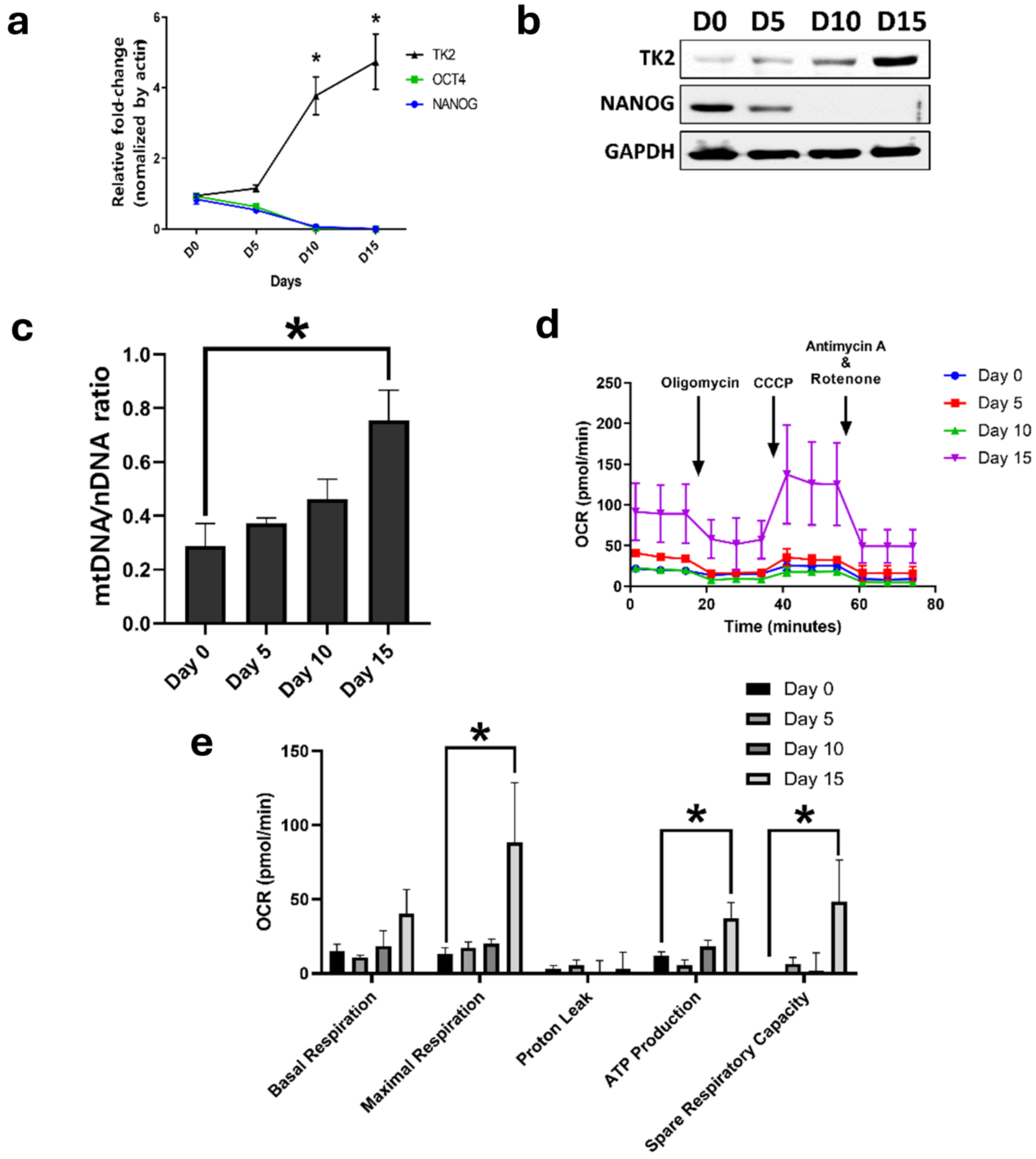

Supplementary Fig. 2

Alterations in TK2 expression and mtDNA copy number and mitochondrial function during embryoid body differentiation of embryonic stem cells. (a) RT-qPCR analysis of TK2 expression during cell differentiation. (b) Western blot analysis of TK2 and NANOG expression during differentiation. (c) Fluctuations in the mtDNA/nDNA ratio during differentiation. (d, e) Modifications in Mito stress test profile and respiratory parameters during

reprogramming. Results represent mean ± SD ($n = 3$). * denotes statistical significance ($^*p < 0.05$, $^{**}p < 0.005$, $^{***}p < 0.0005$, $^{****}p < 0.00005$). Significant differences were evaluated using one-way ANOVA and Tukey's multiple comparison test. Statistical analyses were conducted using GRAPHPAD 8.0.1. mtDNA, mitochondrial DNA; nDNA, nuclear DNA.

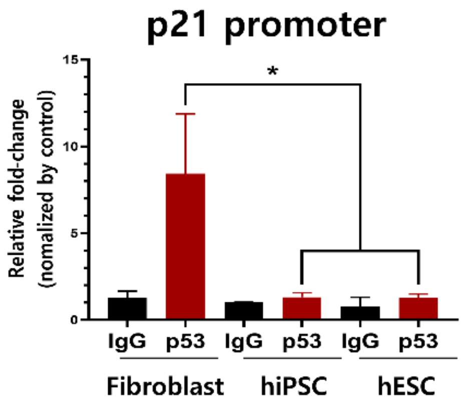

Supplementary Fig. 3

ChIP-qPCR analysis of p53 antibody binding to p21 promoter in fibroblasts and pluripotent stem cells (PSCs).

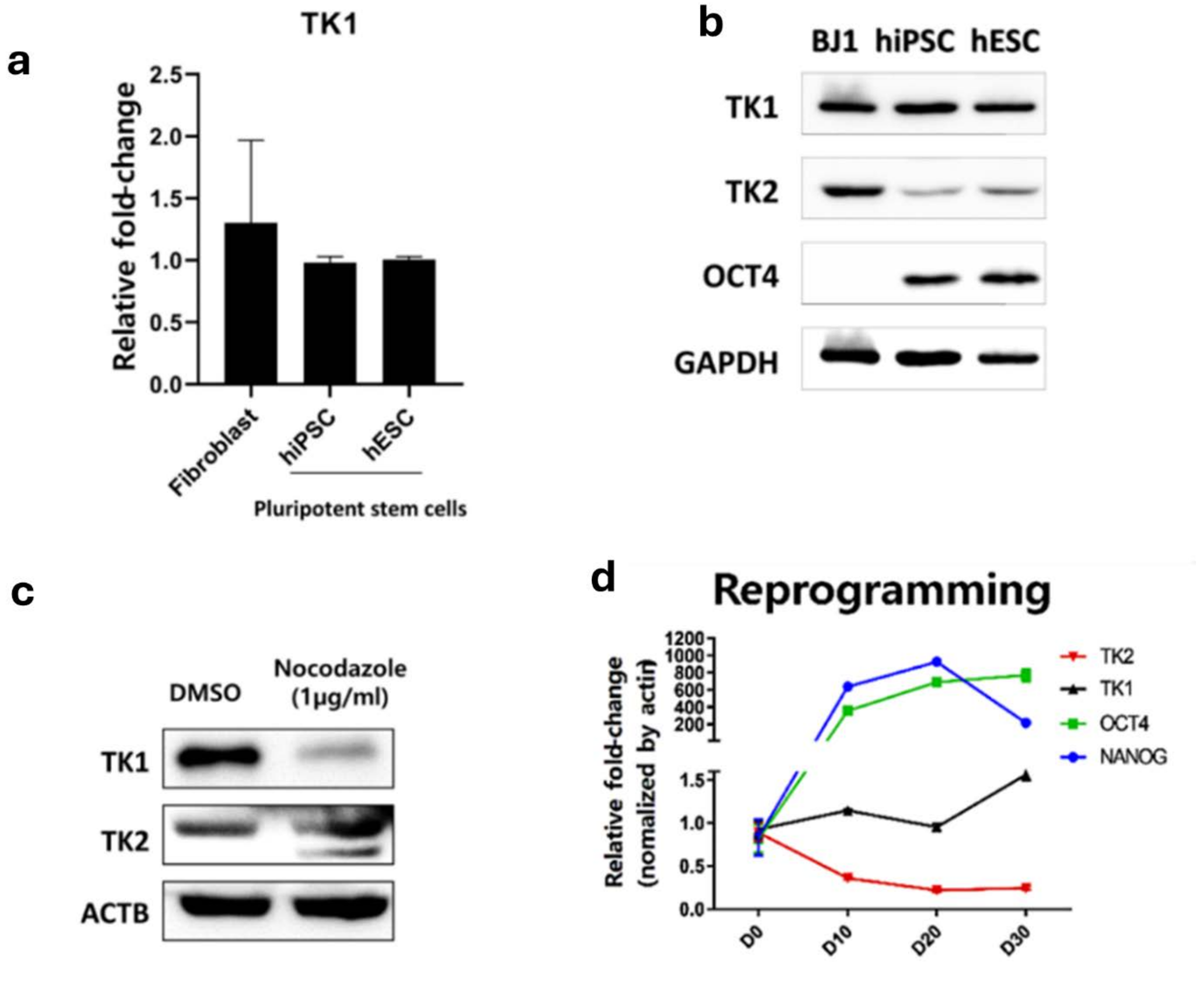

Supplementary Fig. 4

Evaluation of TK1 expression in somatic cells and PSCs and the effect of nocodazole on the cell cycle. (a) RT-qPCR analysis of TK1 expression in fibroblasts and pluripotent stem cells. (b) Western blot analysis of TK1 expression in fibroblasts and pluripotent stem cells. (c) Alterations in TK1 and TK2 protein stability in fibroblasts after 24 h of nocodazole (1 μg/mL) treatment (BJ1). (d) Dynamic changes in TK1, TK2, OCT4, and NANOG expression during somatic cell reprogramming. Relative fold-change in mRNA levels of TK1, TK2, OCT4, and NANOG was assessed at different time points (D0, D10, D20, and D30) during reprogramming.

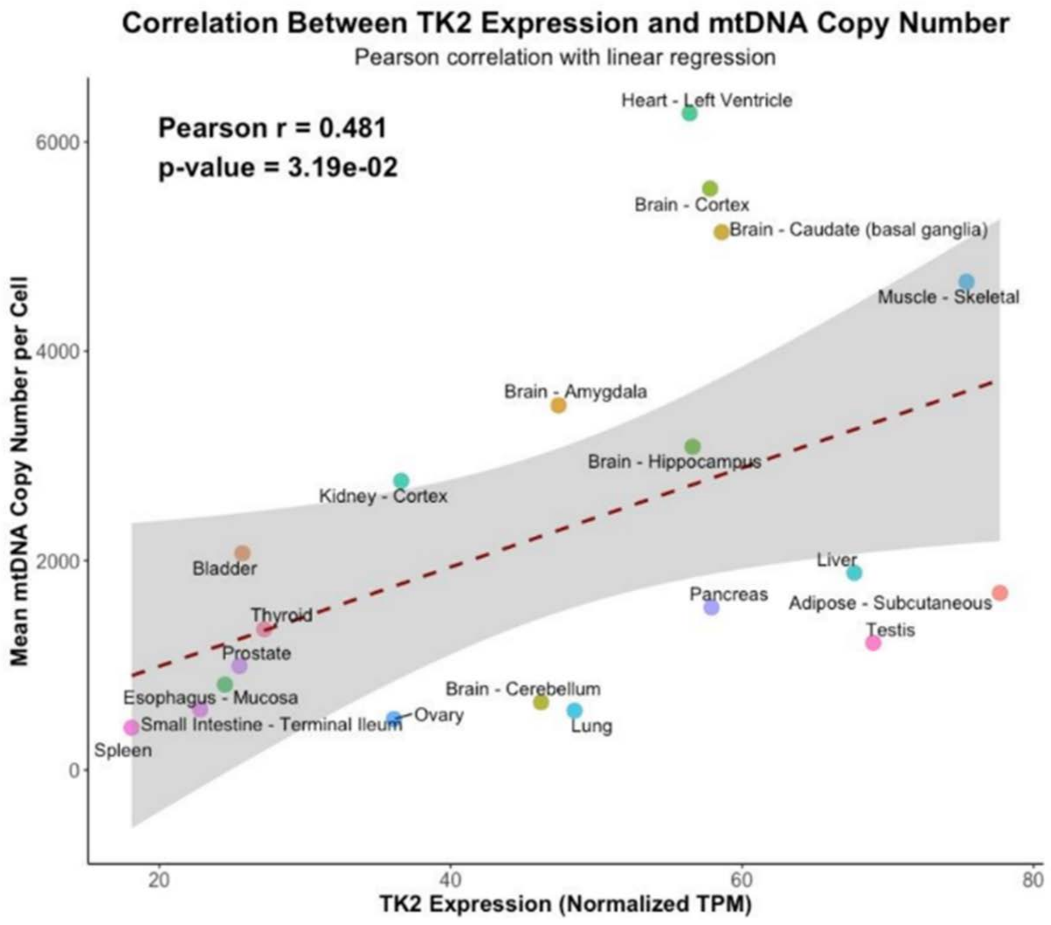

Supplementary Fig. 5

Correlation between TK2 expression and mtDNA copy number.

Scatter plot illustrating the correlation between TK2 expression (x-axis: normalized TPM values from the FANTOM dataset) and mtDNA copy number (y-axis: mean per cell from the PNAS study) across different tissues. The dashed red line represents the linear regression trend, with the gray-shaded area indicating the 95% confidence interval. Each point corresponds to a distinct tissue, which is labeled accordingly. A significant positive correlation was observed (Pearson r = 0.481, p = 0.0319), with metabolically active tissues (e.g., skeletal muscle, heart, and brain tissue) exhibiting higher TK2 expression and mtDNA copy numbers than tissues with lower metabolic activity.

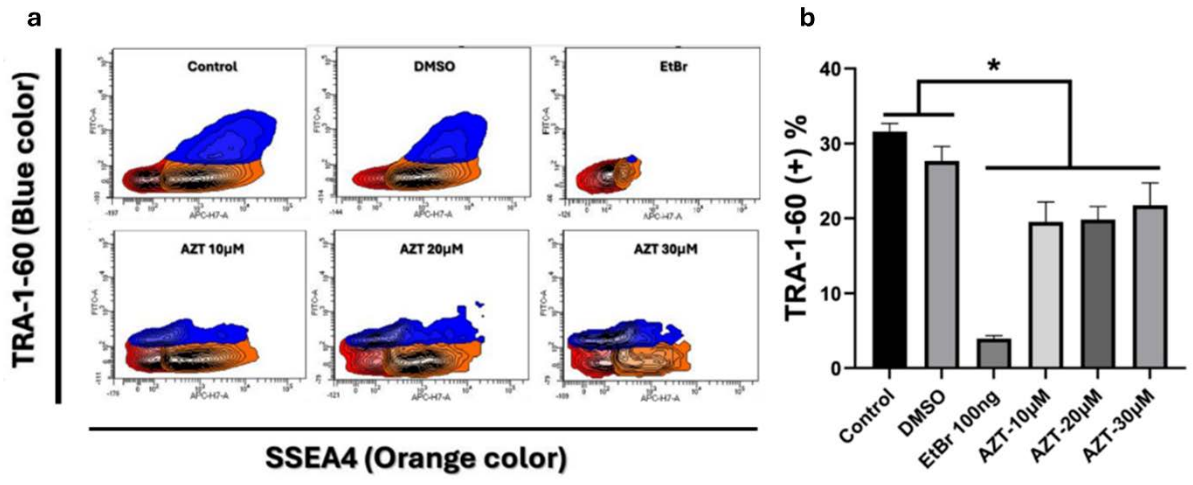

Supplementary Fig. 6

Effect of mtDNA depletion and TK2 inhibition on reprogramming efficiency. (a) Flow cytometry analysis of TRA-1-60 (blue) and SSEA4 (orange) expression under different treatment conditions: control, DMSO, ethidium bromide (EtBr), and AZT (10, 20, and 30 μM). (b) TRA-1-60-positive cell percentage across different treatment conditions. The asterisks indicate statistical significance.
